# First Attempt to Implement Ophthalmia Neonatorum Prophylaxis in Angola: Microorganisms, Efficacy, and Obstacles

**DOI:** 10.1155/2015/326526

**Published:** 2015-02-16

**Authors:** Isabel Alexandre, Mar Justel, Prudencio Martinez, Raul Ortiz de Lejarazu, J. Carlos Pastor

**Affiliations:** ^1^IONA, Eye Institute, Rua de Olivença, Luanda, Angola; ^2^Department of Microbiology and Immunology, Hospital Clinico Universitario, Avenida Ramon y Cajal 3, 47005 Valladolid, Spain; ^3^IOBA, Eye Institute, University of Valladolid, Paseo de Belen 17, 47011 Valladolid, Spain

## Abstract

*Purpose*. To determine the efficacy of povidone-iodine (P-I) prophylaxis for ophthalmia neonatorum (ON) in Angola and to document maternal prevalence and mother-to-child transmission rates. *Methods*. Endocervical samples from mothers (*n* = 317) and newborn conjunctival smears (*n* = 245) were analysed by multiplex polymerase chain reaction (PCR) for *Chlamydia trachomatis* (CT), *Neisseria gonorrhoeae* (NG), and *Mycoplasma genitalium* (MG). Newborns were randomized into a noninterventional group and an interventional group that received a drop of P-I 2.5% bilaterally after conjunctival smear collection. Mothers were trained to identify signs of ON and attend a follow-up visit. *Results*. Forty-two newborns had ocular pathology, and 11 (4.4%) had clinical signs of ON at the time of delivery. Maternal PCR was positive for MG (*n* = 19), CT (*n* = 8), and NG (*n* = 2). Six newborns were positive for CT (*n* = 4), MG (*n* = 2), and NG (*n* = 1). Mother-to-child transmission rates were 50% for CT and NG and 10.5% for MG. Only 16 newborns returned for follow-up. *Conclusions*. Lack of maternal compliance prevented successful testing of prophylactic P-I efficacy in ON prevention. Nevertheless, we documented the prevalence and mother-to-child transmission rates for CT, NG, and MG. These results emphasize the need to develop an effective Angolan educational and prophylactic ON program.

## 1. Introduction

Acute ophthalmia neonatorum (ON) continues to play an important role in causing severe ocular problems of newborns in countries where primary health care coverage is insufficient, as in the case of Angola [1]. Unfortunately, in most clinical obstetric wards of Luanda, the nation's capital, there is no regular plan of ON prophylaxis, nor is there any capacity for determining the microorganisms involved in the etiology of this entity. Therefore, most of the acute conjunctivitis cases in the newborns are empirically diagnosed as ON and treated without having information on the causative agent.

Although prenatal screening and treatment of pregnant women are very effective for the prevention of ON [2], this approach can be difficult to implement in developing countries. In some African countries, at the time of delivery, a large percentage of expectant mothers have had little or no prenatal care.

In industrialized countries, ON prophylaxis was performed by silver nitrate eyedrops and currently by topical erythromycin, tetracycline ointment, and others [2]. In other developed countries, emphasis is placed on maternal surveillance, and there is no systematic prophylaxis of ON.

The use of povidone-iodine (P-I) has been advocated for the less developed countries [3], but few well-designed prospective, randomized, and controlled clinical studies have been performed with this agent [2]. Even while doubts remain regarding the real efficacy of P-I for prevention of ON caused by either* Chlamydia trachomatis* (CT) or* Neisseria gonorrhoeae* (NG) [4, 5], this substance is well tolerated, has a broader spectrum than other anti-infectious agents, does not induce resistance, and is more effective than some antibiotics that are more expensive for prophylaxis [6, 7].

After the civil war in Angola (1974–2002), the public health system was almost completely devastated. In 2009, we conducted a pilot study structured as a maternity ward prospective series and found that 12% of the newborns had signs of acute bilateral conjunctivitis [8]. The problem was considered very serious because there was no program for ON prophylaxis. An additional problem was the inability to determine the microorganisms involved [9–11] because microbiological studies were not routinely performed at either maternity wards or eye centers.

Based upon these existing conditions, we decided to evaluate different tests that could provide necessary microbiological information to the ophthalmologists and then select the best one for implementation in a nationwide ON prophylaxis program. Our initial attempt was to use the simplest available diagnostic tool, standard staining of conjunctival swabs by Giemsa and Gram stains [12]. However, this provided little useful information regarding the active ON agent(s). Following this, we attempted to use multiplex polymerase chain reaction (PCR) of maternal endocervical and neonatal conjunctival specimens for microorganism identification. The PCR assay has been previously validated with conjunctival and endocervical samples collected from this study (manuscript accepted). In brief, we demonstrated that this technique could be used to identify the three major microorganisms, for example, CT, NG, and* Mycoplasma genitalium* (MG), involved in sexually transmitted infections (STIs). This was successful not only in endocervical samples, but also in conjunctival smears, where it never had been used. Therefore, our recommendation for Angola's health authorities was to provide this kind of microbiological identification technique. The primary purpose of this work was to analyze the efficacy, limitations, and obstacles of 2.5% P-I eyedrops for the prophylaxis of ON in Angola. These data are intended to provide the local health authorities with enough information to develop a national prophylactic campaign against ON based on the routine use of P-I eyedrops for every newborn. A secondary objective was to provide additional data on the etiological agents both in conjunctival smears from newborns and in endocervical samples from their mothers and the possible relationship of these agents with clinical risk factors.

## 2. Material and Methods

An interventional, randomized, and prospective study with a blinded, randomized control group was designed. The study was conducted at the General Augusto N'Gangula Specialized Hospital (HGEAG) and the Health Center of Samba (CSS), both in Luanda, Angola. Approval was provided by the Ethical Commission of the Faculty of Medicine of the Agostinho Neto University (Luanda, Angola). After the explanation of the objectives and methodology of the investigation, informed consent was obtained from the mothers. The study was performed in accordance with the ethical standards from the 1964 Declaration of Helsinki and its later amendments.

### 2.1. Patients

The target population for this study consisted of 317 mothers and their newborns from the HGEAG (173) and the CSS (144), recruited from 7 December 2011 to 22 November 2012. The inclusion criteria consisted of healthy children weighing at least 2.3 kg and a gestation period of at least 37 weeks. Newborns not meeting the inclusion criteria and those with respiratory distress at birth were excluded. Additionally, because of the possible deleterious effect of iodine, newborns were excluded if their mothers were diagnosed with thyroid disease according to clinical data contained in her medical record.

### 2.2. Procedure

Maternal data were collected through a questionnaire that included age, race, education, parity, number of prenatal visits, pathology during pregnancy, and duration of rupture of the amniotic sac. Newborn data included weight, Apgar rate, and delivery procedure. Neonates were randomly distributed into two groups, A and B, by blocked randomization with a fixed block size of 4. Newborns in Group A received no intervention. Newborns in Group B received instillation of a drop of P-I 2.5% in the bottom of the lower sac of both eyes immediately after a basic eye examination and the collection of conjunctival smears within 3 hours of birth. Custom 2.5% P-I eyedrops were prepared by a certified Spanish pharmacy (Carreras, Barcelona, Spain) following the standards of good manufacturing practices. For each newborn, a new bottle of P-I was used. Maternal endocervical samples were obtained after removing postpartum secretions from the endocervical os. Ocular samples were obtained from both eyes of the newborns by vigorous swabbing across the inferior tarsal conjunctiva. The collected samples from each eye were then pooled for analysis. All samples were taken by ophthalmologists and medical personnel previously trained in these procedures by an expert microbiologist from Spain (PM).

Samples were collected with flocked swabs in Universal Transport Medium (Copan Italia S.p.A., Brescia, Italy), stored at −70°C, and shipped to the Department of Microbiology and Immunology at the Hospital Clínico Universitario of Valladolid, Valladolid, Spain. DNA extraction was performed according to routine laboratory standards with the GXT DNA/RNA reagents in a GenoXtract extractor (Hain Lifescience, Nehren, Germany). A multiplex PCR assay that coamplified DNA sequences of CT, NG, MG, and an internal control was performed using the Bio-Rad Dx CT/NG/MG Kit (Bio-Rad, Hercules, CA, USA), according to manufacturer's instructions. The ophthalmologist responsible for the study (IA) administered the P-I eyedrops. The basic eye examination given to all newborns included pupil light responses, eyelid position and movement, appearance of the conjunctiva, corneal size and transparency, iris appearance and symmetry, lens transparency, quality and symmetry of retinal red reflex (Brückner test), and the size, position, and shape of the pupil. An information sheet with explanations about the signs of ophthalmia/acute conjunctivitis (red eye and ocular secretions and/or eyelid edema) was given to all participating mothers. They were also given instructions to return with their children for observation after discharge from the maternity ward in any case, even if their baby did not show these signs. The mobile phone number of each mother was noted (only two mothers gave no indication of telephone contact). Between the 5th day and 7th day postpartum, phone calls were made to the mothers to bring their infants for observation, especially if they presented ocular signs of ON or any other ocular alterations.

The minimum sample size was calculated taking in consideration the suspected prevalence of ON detected in our previous study [8] and with an expected reduction of ON in at least 30% in the prophylaxis group (Group B). To detect a 8% difference between treatment groups with a significance level of 5% and power of 80%, a sample size of 334 newborns was estimated, 167 per group. Infection rates of the different ON bacteria in the endocervical samples of the mothers and in the conjunctival samples of their newborns and the presence of clinical risk factors were compared by *χ* ^2^ test.

## 3. Results

The mean age of the 317 participating mothers was 25 years (range: 14–52 years), with the majority between 14 and 24 years. Some of the mothers, 28%, had a basic level of education and 2.2% were illiterate and the remainders have good reading skill. Parity varied from 0 to 9 births, with 82% of the mothers having had 3 previous deliveries. There was no parity data for 4 cases. The number of prenatal consultations of the mothers ranged from 0 to 9, with an average of 4 consultations. Thirty-eight cases (1.2%) did not have any prenatal consultation, and 112 cases (35%) had less than four. There was no data for prenatal consultations from 19 cases. A total of 96 mothers (30.4%) referred some pathology during pregnancy, predominately urinary infection in 81 mothers (25.6%) and vulvovaginitis in 13 others (4.1%). A total of 70 mothers (22%) presented with premature rupture of membrane (PROM). Thirty-one of them had more than 6 hours before delivery, and 39 had less than 6 hours. Data were collected from 317 children, but a total of 72 newborns were excluded (22.6%) from the study for low weight, respiratory distress, death, or transfer of the mother to a more specialized center for dystocic delivery (Table 1). Newborns, 123 females and 118 males, had a gestational age of 36 to 40 weeks, and an average weight of 3.260 kg. The sex of four newborns was not registered. The predominant mode of delivery was vaginal (88.6%). A total of 42 (17.1%) had ocular pathology at the time of delivery, including 31 (12.6%) with conjunctival hyperemia and 11 (4.4%) with ON signs, including conjunctival hyperemia plus eyelid edema and/or purulent secretion. All newborns with suspected ON were delivered vaginally. Three out of the 11 suspected ON cases were born from mothers with urinary infections during pregnancy. For five of the newborns with signs of ON, the amniotic sac did not rupture prematurely. For the other six, there was no data on the time of sac rupture.

Due to technical difficulties in the preparation of the samples for transport to Spain for PCR analysis, there was no information available regarding the presence or absence of CT, MG, and NG for 6 maternal endocervical samples and 13 newborn conjunctival smears. A total of 543 valid samples were analyzed, 232 from conjunctival smears and 311 from endocervical samples (Table 2). The most common etiologic agent in newborns was CT (*n* = 4), followed by MG (*n* = 2) and then NG (*n* = 1). PCR gave positive results in 28 mothers, with a predominance of MG (*n* = 19, 6.1%), followed by CT (*n* = 8, 2.1%) and NG (*n* = 2, 0.5%). One endocervical sample was coinfected with CT and MG. Eleven of the 28 mothers (39.3%) who were infected with CT, MG, or NG presented risk factors for mother-to-child transmission. The factors included PROM at delivery time (*n* = 6, all positive for MG), vulvovaginitis (*n* = 3), urinary tract infection (*n* = 1), and urinary tract infection plus vaginitis (*n* = 1). Of the cases with PROM with >6 hours of labor, one had NG and 3 had MG. Four mothers with PROM < 6 hours were positive for MG. The mother-to-child transmission rates were 50% for both CT and NG and 10.5% for MG (manuscript accepted). Chi-square analysis showed no significant correlation between cases with external signs of acute conjunctivitis and the presence of urinary or vaginal infections in the mother at the time of delivery. The newborns were randomly distributed into Group A, in which the newborns received no ocular prophylactic treatment (*n* = 130), and Group B, in which the newborns received P-I prophylaxis in both eyes (*n* = 115). Despite the efforts made with every mother to perform a follow-up visit within 7 to 10 days after delivery, only 16 children were evaluated, nine from Group A and seven from Group B. Ten children, 7 from Group A (controls) and 3 from Group B (P-I treated), did not show any ocular pathology. Two children from Group B had a bilateral acute conjunctivitis since birth. Two (one from each group) had acute bilateral conjunctivitis after the third day postpartum. One from Group A had a small conjunctival hemorrhage in one eye, and another, from Group B, had jaundice of both conjunctivas.

## 4. Discussion

The nearly complete disappearance of ON in developed countries has been the result of a combination of factors, including prophylactic measures and, above all, better prenatal care [1–3, 11, 13]. However, currently in Angola and other West African nations, no prophylactic measures are used, and it is routinely very difficult to identify the pathogenic agents. Thus, diagnosis of ON is based on clinical signs [8], and systematic follow-up of children after birth is almost impossible.

NG and CT are currently the most common ON etiologic agents, accounting for 60% of all cases in countries without neonatal prophylaxis [14–16]. Although gonococcal infection is less common in developed countries, it continues to be a problem in developing countries such as Kenya where ON has an incidence as high as 4% of live births for NG and 8% for CT [13, 16].

ON due to NG can infect the fetus by ascending from the vagina to the uterus and can be present in the newborn at birth as an acute conjunctivitis; however, other agents usually have an incubation period of 4–14 days before clinical signs [13]. Therefore, clinical conjunctivitis often develops after children are discharged from the maternity ward.

The aim of this study was to evaluate the efficacy of intervening at birth by providing P-I as a prophylactic agent for ON. The weak response of mothers returning for a second ophthalmic examination for their newborns prevented the achievement of this goal. Maternal ignorance about the implications of ON and cultural factors resulted in poor cooperation of mothers in the follow-up evaluations. Even after careful oral and written explanations were given to mothers about the signs and symptoms of ON and the ocular and systemic risk for the affected children, and even after most of the mothers were personally contacted by mobile phone, only a very small number of children, 5.7% of the global sample, returned for a second examination. These kinds of problems have been recognized by other authors [6, 7, 14].

Since 1990, P-I has been considered as a potential prophylactic agent of ON [15]. Several studies have established that P-I has broad spectrum of action and is effective against most agents of ON, unlike other prophylactic agents previously used [16–21]. P-I does not induce bacterial resistance, has low toxicity, is of very low cost, and is stable for several months after opening. Even more, the transient brownish staining of the ocular surface after instillation could be useful as an indicator of its effective application. In 2002, Isenberg reported that 2.5% P-I was not irritating to the eye [19]. Overall, the studies showed that when trained personnel applied P-I after hygienic and general care of the newborn, the results are superior to the other prophylactic agents [16, 19]. Nevertheless, a number of arguments have been raised against its use as a prophylactic agent, including the possibility that it could be confused with a detergent, the lack of effect of P-I against viruses, and lack of studies proving that it is safe in newborns [15, 19].

Our study also shows for the first time the frequency of MG infection and some associated clinical findings in a cohort of Angolan mothers and their newborns (manuscript accepted). MG is an emerging cause of STIs and has been implicated in urogenital infections of men and women worldwide [22]. MG has a prevalence of 7.3% and 2% in high- and low-risk populations, respectively [23]. It is considered as an etiologic agent of pelvic inflammatory diseases and infertility. There is also evidence that this microorganism has the potential to cause ascending infection and may play an important role in the ON [22, 23].

Besides the fact that ON is a potential cause of blindness, it can also result in serious systemic complications when nasopharyngeal colonization during vaginal delivery evolves to otitis, pneumonitis arthritis, sepsis, and meningitis [13, 15, 24]. Risk factors associated with ON are genitourinary infection and PROM [10, 13, 15]. In this study, a total of 108 mothers (34.1%) presented some of these risk factors. According to World Health Organization recommendations, prenatal care of mothers should include identification and management of infections including HIV, syphilis, and other STIs. Regular screening for STIs is not routine in prenatal care in Angola although the majority of mothers included in this study did have the prenatal appointments recommended by WHO [25]. Most of them had 4 or more prenatal visits, and the percentage of mothers without prenatal care was considerably lower than the one obtained by our group in the same maternity ward three years before (1.2% versus 14.7%) [12], showing an improvement of the health system of the country.

Prenatal studies in mothers of similar age as in this study are very important because the age group between 15 and 25 years of age is considered to be at risk of STIs. Actually, genital CT (serotype D-K) and NG infections are especially common in this age group of African women; therefore, their neonates are also a risk group for ON and systemic complications [10, 13, 15]. Our data show that the prevalence of CT in our population was relatively low when compared to previous studies in Angola and other African populations [26]. Other studies of pregnant women have shown prevalence rates from about 6% in Tanzania to 13% in Cape Verde [27]. The prevalence in our study was much lower, which confirms the relatively low presence of CT in Angola.

NG and CT infections have common clinical features in women. Both produce silent clinical infections, are transmitted efficiently to newborns, and can lead to sterility or chronic infection. Both generate silent carriers, causing severe clinical consequences over time [28]. According to historical data, around 3% of newborns with NG ophthalmia will develop complete blindness if untreated, and 20% will have some degree of corneal damage [27]. In Africa, the prevalence rate of NG among pregnant women ranges from 0.02% in Gabon to 7.8% in South Africa [29]. Our results showed a lower prevalence of NG, 0.5%, among mothers, confirming our previous results (manuscript accepted). The results of the present study also allow us to estimate the rates of mother-to-child transmission which were 50% for CT and NG and 10.5% for MG. To our knowledge, this is the first study that estimates the rate of MG transmission in Angola.

In our cohort, the majority of newborns were born vaginally, a risk factor for ON [9]. A total of 12 newborns presented signs of acute conjunctivitis at the first ophthalmic evaluation, although laboratory tests were positive for MG only in one case. On the other hand, there were six cases that did not show signs of acute conjunctivitis at the first observation but were nevertheless positive for CT, MG, or NG.

Neonatal infections are of great epidemiological importance, so focused screening efforts should be made to reduce the number of infected pregnant women and thereby the rate of vertical transmission. To a similar extent as seen for other major STIs in Africa [30], our work clearly shows a mirror effect when considering matches between infected newborns and their mothers. This result also indicates the need to locally improve public information about primary health care particularly that oriented to eye care. Without this knowledge, the participation of community members in studies like ours will not be effective.

In summary, we determined the prevalence and incidence of CT, NG, and MG, which are all implicated in Angolan cases of newborn ON. We also determined that the mother-to-child transmission rates of CT and NG were 50% and 10.5% for MG, which was identified for the first time in Angolan newborns. Unfortunately, due to low compliance in follow-up clinical assessments, we were unable to achieve the original goal of testing the efficacy and safety of P-I systematic prophylaxis for preventing ON.

## Figures and Tables

**Table 1:** Summary of the cases and samples.

Maternity	Mothers included	Newborns included	Endocervical samples analysed	Conjunctival samples analysed	Newborns with signs of acute conjunctivitis at the delivery	Newborns treated with povidone-iodine	Follow-up examination
Augusto N'Gangula	173	140	171	108	20	57	14^*^
Samba	144	105	140	75	22	58	2

Total	317	245	311	183	42	115	16

^*^Four children had signs of acute conjunctivitis with discharge at the follow-up visit.

**Table 2:** PCR-based diagnosis of infectious agents.

	*n*	Cases with mother and newborn infected by same microorganism	Coinfections
Total valid newborn conjunctival samples	232		
CT	4		
NG	1		
MG	2		
Missing samples	13		
Total valid maternal endocervical samples	311		
CT	8	2	1
NG	2	1	
MG	19	1	1
Missing samples	6		

CT: *Chlamydia trachomatis*. NG: *Neisseria gonorrhoeae*. MG: *Mycoplasma genitalium*. Missing data were due to the inability to run PCR samples. Coinfections: one mother had CT and MG at the same sample.

## References

[1] Ayena K. D., Amedome K. M., Diallo J. W. (2012). What remains today of neonatal conjunctivitis in prefecture of Kozah in Togo?. *Journal Francais d'Ophtalmologie*.

[2] Hammerschlag M. R. (2011). Chlamydial and gonococcal infections in infants and children. *Clinical Infectious Diseases*.

[3] Benevento W. J., Murray P., Reed C. A., Pepose J. S. (1990). The sensitivity of *Neisseria gonorrhoeae*, *Chlamydia trachomatis*, and herpes simplex type II to disinfection with povidone-iodine. *American Journal of Ophthalmology*.

[4] Ramirez-Ortiz M. A., Rodriguez-Almaraz M., Ochoa-DiazLopez H., Diaz-Prieto P., Rodriguez-Suárez R. S. (2007). Randomised equivalency trial comparing 2.5% povidone-iodine eye drops and ophthalmic chloramphenicol for preventing neonatal conjunctivitis in a trachoma endemic area in Southern Mexico. *British Journal of Ophthalmology*.

[5] David M., Rumelt S., Weintraub Z. (2011). Efficacy comparison between povidone iodine 2.5% and tetracycline 1% in prevention of ophthalmia neonatorum. *Ophthalmology*.

[6] Simon J. W. (2003). Povidone-iodine prophylaxis of ophthalmia neonatorum. *British Journal of Ophthalmology*.

[7] Isenberg S. J., Apt L., Wood M. (1995). A controlled trial of povidone-iodine as prophylaxis against ophthalmia neonatorum. *New England Journal of Medicine*.

[8] Pastor J. C., Alexandre I. Development of a national prophylactic program against neonatal conjunctivitis in Angola: pilot study.

[9] Schaller U. C., Klauss V. (2001). Is Credé's prophylaxis for ophthalmia neonatorum still valid?. *Bulletin of the World Health Organization*.

[10] Amini E., Ghasemi M., Daneshjou K. (2008). A five-year study of ophthalmia neonatorum in Iran: prevalence and etiology. *Medical Science Monitor*.

[11] Ghahramani M., Ghahramani A. A. (2007). Epidemiologic study of ophthalmia neonatorum and impact of prophylaxis on its incidence. *Acta Medica Iranica*.

[12] Alexandre I., Cortes N., Justel M., Fernández I., Ortíz de Lejarazu R., Pastor J. C. (2014). The value of simple microbiological studies for on-site screening of acute neonatal conjunctivitis in Angola. *Journal of Ophthalmic Inflammation and Infection*.

[13] Hammerschlag M., Rapoza P. (1996). Neonatal conjunctivitis. *Ocular Infection Immunity*.

[14] Isenberg S. J., Apt L., del Signore M., Gichuhi S., Berman N. G. (2003). A double application approach to ophthalmia neonatorum prophylaxis. *British Journal of Ophthalmology*.

[15] Passos A., Agostini F. (2011). Conjuntivite neonatal com ênfase na sua prevenção. *Revista Brasileira de Oftalmologia*.

[16] Kramer A., Aspock C., Assadian O. (2002). Prophylactic indications for eye antiseptics. Prophylaxis against ophthalmia neonatorum. *Developments in Ophthalmology*.

[17] Bell T. A., Grayston J. T., Krohn M. A. (1993). Randomized trial of silver nitrate, erythromycin, and no eye prophylaxis for the prevention of conjunctivitis among newborns not at risk for gonococcal ophthalmitis. Eye Prophylaxis Study Group. *Pediatrics*.

[18] Bell T. A., Sandstrom K. I., Gravett M. G. (1987). Comparison of ophthalmic silver nitrate solution and erythromycin ointment for prevention of natally acquired chlamydia trachomatis. *Sexually Transmitted Diseases*.

[19] Isenberg S. J., Apt L., Valenton M. (2002). A controlled trial of povidone-iodine to treat infectious conjunctivitis in children. *American Journal of Ophthalmology*.

[20] Isenberg S. J., Apt L., Yoshimori R., Leake R. D., Rich R. (1994). Povidone-iodine for ophthalmia neonatorum prophylaxis. *The American Journal of Ophthalmology*.

[21] Vedantham V. (2004). Prophylaxis of ophthalmia neonatorum. *British Journal of Ophthalmology*.

[22] Sethi S., Singh G., Samanta P., Sharma M. (2012). Mycoplasma genitalium: an emerging sexually transmitted pathogen. *Indian Journal of Medical Research*.

[23] McGowin C. L., Anderson-Smits C. (2011). Mycoplasma genitalium: an Emerging Cause of Sexually Transmitted Disease in Women. *PLoS Pathogens*.

[24] Allen U., Davies H. D., Embree J. (2002). Recommendations for the prevention of neonatal ophthalmia. *Paediatrics and Child Health*.

[25] Lincetto O., Mothebesoane-Anoh S., Gomez P., Munjanja S., Joy L., Kate K. (2006). Antenatal care. *Opportunities for Africa's Newborns: Practical Data, Policy and Programmatic Support for Newborn Care in Africa*.

[26] Cappuccinelli P., Gomes E., Rubino S. (1995). Chlamydia trachomatis in gynaecological infections in Luanda, Angola. *Genitourinary Medicine*.

[27] WHO (2001). *Global Prevalence and Incidence of Selected Curable Sexually Transmitted Infections: Overview and Estimates*.

[28] McNeeley S. G. (1989). Gonococcal infections in women. *Obstetrics and Gynecology Clinics of North America*.

[29] Gilbert C., Foster A. (2001). Childhood blindness in the context of VISION 2020—the right to sight. *Bulletin of the World Health Organization*.

[30] de Cock K. M., Fowler M. G., Mercier E. (2000). Prevention of mother-to-child HIV transmission in resource-poor countries: translating research into policy and practice. *The Journal of the American Medical Association*.

